# Special Issue “Recent Advances of Natural Products in Chemical and Biological Aspects”

**DOI:** 10.3390/ijms27114878

**Published:** 2026-05-28

**Authors:** Francisco Les, Marta Sofía Valero, María Pilar Arruebo

**Affiliations:** 1 Department of Pharmacy, Faculty of Health Sciences, Universidad San Jorge, 50830 Zaragoza, Spain; 2 Instituto Agroalimentario de Aragón-IA2, CITA-Universidad de Zaragoza, 50013 Zaragoza, Spain; msvalero@unizar.es (M.S.V.); parruebo@unizar.es (M.P.A.); 3 Departamento de Farmacología, Fisiología y Medicina Legal y Forense, Universidad de Zaragoza, 50009 Zaragoza, Spain

## 1. Introduction

Natural products keep earning their place in molecular science for a simple reason: they are chemically inventive, biologically “opinionated”, and often arrive with a legacy of pre-existing relationship to human use that invites hypotheses instead of guesswork [1]. Unlike synthetic libraries, these compounds are the result of evolutionary co-selection, meaning they occupy a biologically relevant chemical space that synthetic chemistry is only beginning to map [2,3,4]. However, this evolutionary head start is no longer a free pass in modern pharmacology.

In an era defined by precision and high-resolution data, the study of natural compounds has moved beyond the mere observation of activity to demand a rigorous mapping of their interaction with the human proteome [5], signaling networks [6], and even the symbiotic microbiota [7,8]. We are moving from a descriptive era, where we observe what a plant does, to a mechanistic era, where we decipher how it communicates with our cellular machinery. Yet, the bar has risen [9]. Today, demonstrating activity is rarely enough; reproducibility, target plausibility, pathway mapping, and model relevance decide whether a study becomes a useful reference or just a fleeting signal [10,11].

This Special Issue brings together seven contributions that collectively illustrate this shift: from ethnopharmacological context and compositional rationale to mechanistic experimentation, multi-omics readouts, host-microbiota connections, and direct biophysical interrogations of antiviral targets. In other words, it is not a catalog of “natural things that help”, but a snapshot of how natural-product research is evolving when authors aim to explain, not merely to report.

What follows is a thematic tour of the papers, grouping them by biological question and translational logic rather than by publication date.

## 2. Ethnopharmacology as a Hypothesis Engine: From Tradition to Mechanisms

The use of medicinal plants over decades represents a bioactive potential previously screened by the population. Rather than mere tradition, ethnopharmacology acts as a hypothesis engine, where centuries of human observation provide the primary clues for modern drug discovery [12]. By applying rigorous pharmacological standards to these traditional claims, researchers can bypass the randomness of synthetic screening and focus on compounds with established biological relevance [2].

In this context, *Jasonia glutinosa* (L.) DC.: Back in Our Pantries? A Review of Its Pharmacological Activity and Mechanisms of Action [13] serves as a compelling case study on how traditional knowledge can be translated into testable pharmacology. Known across the Iberian Peninsula, southern France, and Morocco as “Rock Tea”, this medicinal plant has long been a staple of folk medicine for treating digestive, analgesic, and respiratory ailments.

In this narrative review, the authors conduct a comprehensive database synthesis to align these historical uses with contemporary experimental evidence. By studying the plant’s phytochemical composition and its bioactivity against in vitro, ex vivo, and in vivo models, the study provides a robust scientific validation of its therapeutic potential. This work is particularly significant because it addresses a common issue in ethnopharmacology: by aligning reported spasmolytic and anti-inflammatory properties with specific molecular pathways, the study provides solid scientific validation that justifies its return to the forefront of modern clinical practice. Ultimately, the work highlights a practical point that resonates throughout this Special Issue: mechanisms are the bridge that turns cultural memory into molecular plausibility.

## 3. Inflammation and Barrier Dysfunction: Lungs, Immunity, and Signaling Pathways

Flavonoids have the potential to ameliorate various lung disorders by significantly strengthening the epithelial barrier and modulating inflammatory cascades, providing a multi-target therapeutic approach for respiratory diseases. Furthermore, these compounds contribute to restoring normal lung function and preventing tissue remodeling, primarily due to their potent antioxidant and anti-inflammatory properties. Therefore, flavonoids can be valuable and beneficial in reducing the development of pulmonary diseases such as COPD, ARDS, asthma, and lung cancer [14,15].

Amelioration of Inflammation in Rats with Experimentally Induced Asthma by Spenceria ramalana Trimen Polyphenols via the PI3K/Akt Signaling Pathway [16] tackles airway inflammation using a rat asthma model induced by ovalbumin plus cigarette smoke. Beyond improvements in inflammatory cell counts and cytokine profiles (including Th2-associated mediators), the study connects efficacy to epithelial barrier integrity (e.g., tight-junction markers) and to downregulation of the PI3K/Akt axis. Notably, the work also extends into the gut-lung axis, reporting changes in microbiota and fecal metabolites alongside respiratory outcomes. This helps the narrative feel “systems-level” rather than organ-isolated.

A complementary immunology angle appears in An Ingenane-Type Diterpene from Euphorbia kansui Promoted Cell Apoptosis and Macrophage Polarization via the Regulation of PKC Signaling Pathways [17]. Here, the natural-product scaffold (an ingenane-type diterpene) is positioned not only as a cytotoxic candidate but as an immune modulator. Macrophage polarization is linked to PKC-related signaling (including the PKC-δ/ERK axis). Read alongside the asthma study, the contrast is compelling: one paper “pulls down” inflammatory circuitry in a barrier disease via PI3K/Akt, while the other “re-wires” immune cell behavior in an oncologic setting via PKC pathways. Different tissues, different stakes, same underlying idea: mechanism matters because it tells you where to intervene next.

## 4. Metabolic Dysfunction and the Host-Microbiota Interface: Functional Ingredients with Molecular Consequences

Observational and mechanistic evidence over the last decades has established that the gut microbiota is a central determinant of human metabolic health, where its dysregulation contributes to the pathogenesis of obesity, type 2 diabetes, and cardiometabolic diseases [18,19].

In this context, Low-Molecular-Weight Bovine Collagen Peptides Reduce Fat Accumulation in C. elegans and Ameliorate Obesity-Related Metabolic Dysfunction and Microbiota Diversity in C57BL/6 Male Diet-Induced Obese Mice [20] sits at the intersection of nutraceutical science and mechanistic metabolism. The methodological rigor of this paper is defined by its dual-model strategy: using C. elegans for high-throughput screening of fat accumulation, oxidative stress, and lifespan-related genes (like daf-16 and sod-3), before moving into the complexity of a murine diet-induced obesity model. This sequential approach reveals that low-molecular-weight peptides act as signaling molecules that improve glucose tolerance and adiposity distribution. The take-home is not just “collagen peptides help”, but that a specific low-molecular-weight preparation can trigger measurable metabolic shifts while coinciding with microbiota remodeling, a theme that echoes the gut-microbiota/metabolite dimensions of the asthma paper, though in a very different disease frame.

## 5. Neuroprotection with Multi-Omics Logic: When Pathway Claims Are Backed by Proteome-Scale Data

Neuroprotection is a particularly demanding field for natural products research because neurodegenerative disorders rarely depend on a single molecular event [21]. Oxidative stress, mitochondrial impairment, neuroinflammation, synaptic dysfunction, altered trophic signaling, and apoptosis often converge in the same pathological process. In this context, omics-based approaches are especially valuable: they allow the biological effect of a compound to be read at the network level before being narrowed down to selected pathways for mechanistic validation. Proteomics, in particular, helps bridge the gap between broad phenotypic improvement and molecular interpretation, provided that global protein changes are followed by targeted confirmation of the most relevant signaling axes [22].

Tandem Mass Tags Quantitative Proteomics Reveal the Mechanism by Which Paeoniflorin Regulates the PI3K/AKT and BDNF/CREB Signaling Pathways to Inhibit Parkinson’s Disease [23] represents this Special Issue’s strongest “systems biology” approach. By employing tandem mass tag proteomics alongside in vitro and in vivo validation, the authors demonstrate paeoniflorin’s capacity to mitigate mitochondrial dysfunction, oxidative stress, and apoptosis in Parkinson’s disease models.

The study’s significance lies in its ability to deconstruct neuroprotective effects at a granular level. The proteomic data reveals that paeoniflorin does not act on a single target but rather orchestrates a broad therapeutic response. By highlighting the PI3K/AKT and BDNF/CREB signaling axes as central mechanistic drivers, the authors provide multi-layered verification: they capture the “broad picture” through proteomic shifts and the “precise detail” via targeted confirmatory assays.

Notably, there is a compelling conceptual link to the asthma study: the PI3K/Akt axis emerges once again, yet here within a neuroprotective context. This serves as a reminder that molecular pathways are conserved “languages” across pathologies, where the specificity lies not in the circuit itself, but in the cell type and the resulting phenotypic endpoint.

## 6. Antiviral Targeting Beyond Generic “Activity”: Engaging a Viral Structural Protein

Natural products with antiviral potential are often first described through broad reductions in viral infectivity, replication, or cytopathic effect. While such evidence is essential, the maturation of the field increasingly depends on moving beyond the general concept of “antiviral activity” toward the identification of defined molecular targets within the viral cycle [24]. Structural proteins are particularly relevant in this regard, as they participate not only in virion architecture but also in assembly, trafficking, budding, and host–cell interactions. Targeting these proteins with bioactive molecules may therefore reveal antiviral mechanisms that are distinct from classical inhibition of viral enzymes and may open new opportunities for compounds traditionally considered pleiotropic or nonspecific [25].

Interaction of Human Respiratory Syncytial Virus (HRSV) Matrix Protein with Resveratrol Shows Antiviral Effect [26] takes a polyphenol that many readers “already know” (resveratrol) and shifts the conversation toward a more precise question: what does it bind, and why could that matter for viral replication?

The editorial value of this work lies in its departure from purely phenomenological antiviral screening. The study focuses on the RSV matrix (M) protein, which is essential for viral budding. Through biophysical characterization of the M/resveratrol interaction. The authors interpreted binding in relation to the dimerization interface required for oligomer formation. In the landscape of natural products research, this level of target-specific detail is invaluable: it elevates a broadly bioactive compound into a focused hypothesis about a tractable antiviral node.

## 7. Cancer: Resistant Phenotypes, Combinability, and Immune-Context Modulation

A shift in focus regarding anti-tumor strategies is currently underway. Oncology is moving away from monotherapies toward therapies that encompass tumor heterogeneity and the complex ecosystem of the tumor microenvironment. Natural products are increasingly recognized not only as cytotoxic agents but as chemosensitizers capable of targeting resilient cellular subpopulations and modulating the immune context to overcome therapeutic resistance [27]. In this framework, cancer research in this Special Issue is represented by two contributions that, taken together, feel deliberately modern: one targets stem-like tumor subpopulations and their combinability with standard agents, while the other addresses immune-context dynamics.

Inhibition of Cancer Stem-like Cells by Curcumin and Other Polyphenol Derivatives in MDA-MB-231 TNBC Cells [28] addresses triple-negative breast cancer through the lens of therapeutic resistance. This work lies in its focus on the root of tumor relapse. The authors evaluate synthetic derivatives inspired by EGCG and curcumin, finding mammosphere inhibition and the downregulation of stemness-related markers, providing a mechanistic basis for relapse-drug-combination testing. The reported synergy between a curcumin derivative (TL3) and conventional chemotherapy (doxorubicin/cisplatin) is the kind of result that tends to influence what people try next in the lab, because it proposes a pragmatic route to translation: do not replace chemotherapy; make it more effective against the subpopulation most likely to drive relapse.

As noted earlier, the study on the ingenane-type diterpene from *Euphorbia kansui* [23] adds a complementary layer by coupling apoptosis with macrophage polarization linked to PKC signaling. If the TNBC paper is about hitting the “hard-to-kill” cell state, the *Euphorbia* study is about shaping the neighborhood in which tumor cells survive. Side by side, they sketch a broader natural-product strategy in oncology: combining direct cytotoxic or anti-stemness effects with immune-cell pathway modulation, both grounded in signaling logic rather than descriptive endpoints, offering a dual-pronged approach to overcoming tumor resilience.

## 8. Closing Note

Across these contributions, several patterns emerge naturally: a preference for mechanistic anchoring (PI3K/Akt, BDNF/CREB, PKC-related signaling), the adoption of multi-layer validation (proteomics, network pharmacology, microbiota profiling, biophysical binding assays), and a pragmatic approach to translation (combination therapy, barrier integrity, functional-ingredient plausibility). This Special Issue, viewed as a whole, suggests that the field is converging toward a more demanding but more useful standard: natural products as mechanistically accountable bioactives, not just “natural” observations [29].
